# Oropharyngeal geometry and acoustic parameters of voice in healthy and Parkinson's disease subjects

**DOI:** 10.1590/2317-1782/20232021304en

**Published:** 2023-04-17

**Authors:** Joice Maely Souza da Silva, Adriana de Oliveira Camargo Gomes, Maria das Graças Wanderley de Sales Coriolano, Julianne Pitanga Teixeira, Hellen Vasconcelos Silva Leal de Lima, Clarissa Evelyn Bandeira Paulino, Hilton Justino da Silva, Zulina Souza de Lira

**Affiliations:** 1 Programa de Pós-graduação em Saúde da Comunicação Humana, Universidade Federal de Pernambuco - UFPE - Recife (PE), Brasil.; 2 Departamento de Fonoaudiologia, Universidade Federal de Pernambuco - UFPE - Recife (PE), Brasil.; 3 Departamento de Anatomia, Universidade Federal de Pernambuco - UFPE - Recife (PE), Brasil.

**Keywords:** Oropharynx, Voice Quality, Acoustics, Dysphonia, Parkinson Disease

## Abstract

**Purpose:**

to verify whether there are differences in acoustic measures and oropharyngeal geometry between healthy individuals and people with Parkinson's disease, according to age and sex, and to investigate whether there are correlations between oropharyngeal geometry measures in this population.

**Methods:**

40 individuals participated, 20 with a diagnosis of Parkinson's disease and 20 healthy individuals, matched by age, sex, and body mass index. Acoustic variables included fundamental frequency, jitter, shimmer, glottal-to-noise excitation ratio, noise, and mean intensity. Oropharyngeal geometry variables were measured with acoustic pharyngometry.

**Results:**

geometry variables were smaller in the group with Parkinson's disease, and older adults with Parkinson's disease had a smaller oropharyngeal junction area than healthy older adults. Regarding acoustic parameters of voice, fundamental frequency values were lower in males with Parkinson's disease, and jitter values ​​were higher in the non-elderly subjects with Parkinson's disease. There was a moderate positive correlation between oral cavity length and volume, pharyngeal cavity length and vocal tract length, and pharyngeal cavity volume and vocal tract volume.

**Conclusion:**

individuals with Parkinson's disease had smaller glottal areas and oropharyngeal junction areas than healthy individuals. When distributed into sex and age groups, the fundamental frequency was lower in males with Parkinson's disease. There was a moderate positive correlation between oropharyngeal length and volume measures in the study sample.

## INTRODUCTION

Parkinson’s disease (PD) is a chronic disease characterized by degeneration of the compact portion of the substantia nigra in the midbrain, causing the loss of dopaminergic neurons^(1-3)^. Its main motor manifestations include bradykinesia, stiffness, tremor at rest, and postural and gait changes^(3,4)^. Other signs may appear in the course of the disease, such as changes in voice production - e.g., difficulties coordinating breathing/phonation articulation, decreased vocal intensity, hoarseness, raspy voice, increased nasality, and reduced muscle control in laryngeal structures^(5)^.

Changes related to voice production in these individuals are usually analyzed from the phonatory standpoint - i.e., related to the glottal source and the aerodynamic function, with little reference to the vocal tract (VT). Therefore, “filter” interferences are not much considered - such filter modifies vocal fold vibration patterns, as some segments of their structures create obstacles to the soundwave generated in the glottal source^(6)^. Hence, VT cavities, which encompass oropharyngeal structures, are directly related to the resulting speech sound, and the dimensions of these structures impact the quality of voice^(7)^.

It is known that changes suffered by people with PD directly impact their speech and voice^(8)^ and that the voice is produced by the sound originating in the glottis with resonance effects throughout the VT^(6)^. Hence, studying VT dimensions may provide important answers to diagnosing and treating these individuals. For instance, such a study may help identify which VT segments increase or decrease vocal intensity and projection, associating its dimensions with the resulting voice quality.

Moreover, different VT adjustments have been followed up with therapeutic or voice improvement techniques, and its geometry and voice results have been measured, which helped monitor the effects of the techniques on the intended voice quality. This hypothesis corroborates the idea that noninvasive instrumental methods that measure VT and its adjustments in the oropharyngeal region help improve the assessment and follow-up of therapeutic results in voice clinical practice when correlated with voice analysis^(9-11)^.

For instance, the area, volume, and length of different oropharyngeal segments can be analyzed with acoustic pharyngometry (AP)^(9,12)^, whose physical principle is that a sound generated in a tube is echoed back. This echo intensity represents the cross-sectional area of the different constrictions of the tube, and the distance of each constriction is calculated by the time the echo takes to arrive, making it possible to map the whole cavity from the incisors to the glottis^(9,10,13)^.

AP allows the patient to breathe freely during the procedure; it is quick, low-cost (in comparison with other imaging examinations), noninvasive, and does not expose the patient to radiation as other examinations^(14)^. AP was first used in sleep research^(12,15-17)^, but it is still little used in studies in Brazil, with recent voice-related publications^(9,10)^.

The analysis of acoustic parameters of voice ensures greater quality in voice assessment, as it furnishes data that are not purely subjective - i.e., they do not depend exclusively on the evaluator’s auditory experience. It also detects vocal manifestations in subclinical conditions in people with neurological diseases, even helping diagnose these diseases^(18,19)^.

Hence, oropharyngeal geometry measures and acoustic parameters of voice must be verified in healthy and PD subjects, considering their age and sex and the possible correlations between oropharyngeal measures. Such results may contribute to studies aiming to ground the use of AP to diagnose and monitor voice therapy results.

Thus, the objective of this study was to verify whether there are differences in oropharyngeal geometry and acoustic measures between healthy and PD subjects, according to age and sex, and whether the oropharyngeal geometry measures are correlated in this population.

## METHODS

Altogether, 40 individuals aged 50 to 70 years participated in the study - 20 of them diagnosed with PD (10 men and 10 women). Their results were compared with individuals without PD; hence, 20 healthy subjects were included (10 men and 10 women), matched with PD patients for age, sex, and body mass index (BMI). PD participants were recruited at the Neurology Outpatient Center in a University Hospital. The healthy group comprised companions of patients who attended the hospital and the Speech-Language-Hearing Teaching Clinic in the same institution, as well as research subjects’ friends and acquaintances not diagnosed with any neurological or voice changes.

Thus, the study had a convenience sample and was approved by the Human Research Ethics Committee under evaluation report no. 2.524.982. All study participants signed an informed consent form. The inclusion criteria were as follows: patients diagnosed with PD, classified into stages 1, 2, and 3 on the original version of the Hoehn & Yahr scale (HY)^(20)^, with preserved cognition, verified with the Mini-Mental State Examination (MMSE)^(21)^. Stratification into age groups considered people 60 years or older as older adults^(22)^.

Information on laryngeal disease diagnoses was obtained with videolaryngoscopy, before beginning data collection. Only individuals without any laryngeal lesions or malformations were included in the research. Individuals with PD associated with other neurological or psychiatric comorbidities, reported laryngeal surgery, head and neck surgery, smokers, alcohol drinkers, or who had the flu or an allergic reaction (such as rhinitis or sinusitis) at the time of the research were excluded from the study.

Sample characterization demonstrates the homogeneity between the two study groups regarding their age, MMSE results, educational attainment, and BMI (Table 1).

**Table 1:** Sample characterization (n = 40 individuals)

Healthy group (n = 20)	mean (standard deviation)	minimum-maximum values
Age (years) - 13 older adults and 7 adults	61 (5)	50-69
Mini-Mental State Examination	24 (4)	14-30
Years at school	8 (5)	2-12
Body mass index	26 (2)	23-29
Parkinson’s disease group (n = 20)	mean (standard deviation)	minimum-maximum values
Age (years) - 12 older adults and 8 adults	61 (6)	50-69
Mini-Mental State Examination	27 (3)	20-30
Years at school	9 (4)	4-15
Body mass index	25 (2)	21-31
Time with disease (years)	7 (5)	1-20
Stage of the disease (HY)	2 (1)	1-2

**Caption:** HY = Hoehn &Yahr Scale^(20)^

Acoustic analysis was made with voice recordings. Participants were instructed to sit on a comfortable chair at a 90º angle and emit a sustained vowel /ɛ/ for 5 seconds and count from 1 to 10 in their usual voice. Speech tasks were recorded at a 44000 Hz sample rate, with 16 bits per sample. The collection was made with an n3 notebook, Intel^®^ Core™ i3-2348M, using an Andrea PureAudio™ USB-AS external sound card and a Karsect HT-2 headset microphone kept about 4 centimeters away from their mouth at an approximately 45º angle.

Acoustic data were recorded and edited in Voxmetria^®^ software, manufactured by *CTS informática*. The initial and final seconds in the sustained vowel recordings were eliminated to exclude the most irregular parts of the sample, preserving 3 seconds of emission for analysis. Data on fundamental frequency (f0), jitter, shimmer, glottal-to-noise excitation ratio (GNE), and noise were extracted from the vowel /ɛ/ emission, and the mean intensity was obtained from the number count. All parameters were calculated with Voxmetria^®^.

All participants had their oropharyngeal geometry assessed with AP while awake. The acoustic pharyngometer used was manufactured by Eccovision^®^ - Sleep Group Solutions, Florida, which was installed in the laboratory of the institution where this study was conducted, controlling temperature (25 ºC) and noise (below 60 dB SPL) during the examination.

The pharyngometer was automatically calibrated to take VT and oropharyngeal measures^(9,10)^. Participants remained seated on a chair with back support, head and trunk aligned. They were instructed to bite the plastic mouthpiece and seal it with their lips, preventing acoustic escape. The mouthpiece is connected to the pharyngometer on one end, positioned horizontally to the examiner and parallel to the floor. To keep their posture, participants were instructed to gaze at a point in front of them and breathe naturally.

For each measure, the program (software) generated a graph relating the distance (ordinate axis) to the area (abscissa axis), subdivided into three regions: oral (from the incisors to the soft palate), pharyngeal (from the soft palate to the hypopharynx), and laryngeal (glottal region).

Participants were instructed to breathe in naturally for a few moments, always through the nose. Then, in agreement with the researcher, they would breathe out through the mouth - measures were taken at the end of each outbreathing^(9,10)^.

Four measures were taken, shown in four widows on the equipment screen, namely:

- Measures of the oropharyngeal area (recorded in the first two windows): participants were instructed to breathe in through the nose and slowly out through the mouth. Oropharyngeal measures were based on the graphs, characterized as possible calibrating graphs, presented in superposition and maximum percentage of reproducibility, with up to 6% acceptable variance.
- Measure of the oropharyngeal junction (recorded in the third window): participants were instructed to breathe in and out through the nose. This made it possible to identify the oropharyngeal junction, delimited at the end of the oral cavity when the soft palate is lowered.
- Measure of the glottal region (recorded in the fourth window): participants were instructed to breathe in through the nose and perform the Valsalva maneuver, in which individuals shut their nostrils with their fingers and then force the air, closing the glottis. Thus, the end of the pharyngeal cavity was located in the graph, indicating the glottal region.

The data were compiled and presented as measures of central tendency and dispersion. The Shapiro-Wilk test was used to verify the normality of the data series, which determined the comparison tests between the group means (independent t-test or Mann-Whitney test). The variables were correlated with Pearson’s test, interpreted with the following criteria: 0.90 to 1.00 = “Very high”; 0.70 to 0.90 = “High”; 0.50 to 0.70 = “Moderate”; 0.30 to 0.50 = “Low”; 0.10 to 0.30 = “Small”^(23)^. Besides Pearson’s rho, determination coefficients, r^2^, were also presented. The statistical package used was Statistica StatSoft 12, considering significant values at p < 0.05.

## RESULTS

The mean values of oropharyngeal geometry and acoustic parameters of voice are shown in Tables 2, 3, and 4, as well as the comparison between the PD group and the healthy group (HG). Measures in Table 3 were stratified by sex and in Table 4, by age groups.

**Table 2:** Values of oropharyngeal segment geometry measures and acoustic parameters of voice in healthy subjects and Parkinson’s disease patients and results of the comparison between both groups

Oropharyngeal Geometry	Healthy Group (n = 20)			Parkinson’s Disease Group (n = 20)			
	mean (standard deviation)	range	P5-P95%	mean (standard deviation)	range	P5-P95%	p-value
*Lengths*							
OCL (cm)	8.5 (1.1)	6.7-11.0	7.1-10.6	8.4 (0.9)	6.7-10.5	7.1-10.2	*0.79* *
PCL (cm)	6.5 (2.5)	2.2-10.7	3.3-10.7	5.8 (1.8)	2.6-9.4	3.0-9.0	*0.67*ͳ
VTL (cm)	15.0 (2.1)	13.1-17.8	13.2-17.9	14.3 (1.7)	13.1-17.8	13.2-17.9	*0.37* ^ͳ^
*Volumes*							
OCV (cm^3^)	36.7 (11.6)	17.0-60.6	19.7-54.3	34.8 (10.6)	20.2-54.0	21.3-53.8	0.59*
PCV (cm^3^)	13.1 (8.2)	2.4-36.3	3.7-22.0	11.3 (9.1)	2.2-34.6	3.0-29.2	*0.33* ^ͳ^
VTV (cm^3^)	49.7 (15.5)	26.1-81.3	30.1-78.0	46.4 (16)	31.0-88.6	31.1-74.8	*0.39* ^ͳ^
*Areas*							
OJA (cm^2^)	1.5 (1.0)	0.5-3.9	0.7-3.6	0.9 (0.8)	0.4-4.6	0.5-1.5	***<0.01*** ^ͳ^
GA (cm^2^)	1.3 (0.7)	0.3-2.9	0.4-2.6	0.8 (0.5)	0.05-2.9	0.3-1.2	***0.04*** ^ͳ^
Acoustic Parameters of Voice	mean (standard deviation)	range	P5-P95%	mean (standard deviation)	range	P5-P95%	p-value
f0 (Hz)	160.9 (38.8)	111.3-233.4	113.8-232.6	149.9 (43.7)	94.2-239.9	99.6-225.7	0.40*
Jitter (%)	0.41 (0.65)	0.09-2.86	0.1-1.5	0.78 (1.14)	0.10-4.21	0.1-3.3	*0.06* ^ͳ^
Shimmer (%)	6.47 (4.5)	1.8-20.0	1.9-13.7	8.54 (6.0)	3.1-23.3	3.3-19.7	*0.26* ^ͳ^
GNE	0.78 (0.17)	0.40-0.98	0.5-1.0	0.72 (0.20)	0.24-0.95	0.3-1.0	*0.25* ^ͳ^
Noise	1.12 (0.72)	0.33-2.73	0.4-2.4	1.29 (0.89)	0.11-3.40	0.4-3.3	*0.51* ^ͳ^
Mean Intensity (dB)	40.0 (5.6)	31.2-52.0	31.3-48.6	39.5 (4.2)	30.2-45.4	32.4-45.0	0.35*

* Independent t-test - 5% significance level

ͳ Mann-Whitney test - 5% significance level

**Caption:** OCL = Oral Cavity Length; PCL = Pharyngeal Cavity Length; VTL = Vocal Tract Length; OCV = Oral Cavity Volume; PCV = Pharyngeal Cavity Volume; VTV = Vocal Tract Volume; OJA = Oropharyngeal Junction Area; GA = Glottal Area; f0 = Fundamental Frequency; GNE = Glottal-to-Noise Excitation Ratio (Proportion Between Glottal Excitation and Noise); range = Minimum-Maximum Values; cm = Centimeters; Hz = Hertz; dB = Decibels

**Table 3:** Values of oropharyngeal segment geometry measures and acoustic parameters of voice stratified by sex and results of the comparison between healthy individuals and Parkinson’s disease patients

Oropharyngeal Geometry	Males (n = 20)					Females (n = 20)				
	mean (standard deviation)		p-value	P5-P95%		mean (standard deviation)		p-value	P5-P95%	
	HG (n=10)	PD (n=10)		HG	PD	HG (n=10)	PD (n=10)		HG	PD
*Lengths*										
OCL (cm)	8.7 (1.1)	8.8 (0.7)+	*0.87* *	7.2-10.4	8.2-10.0	8.2 (1.1)	8.0 (0.9)	0.58*	7.1-10.2	6.9-9.4
PCL (cm)	6.3 (2.6)	6.2 (2.1)	0.88*	3.8-10.7	3.4-9.2	6.7 (2.4)	5.4 (0.4)	0.14*	3.3-9.7	3.8-7.5
VTL (cm)	15.0 (2.4)	15.0 (2.0)	0.85*	13.2-17.9	13.2-17.9	14.9 (1.9)	13.6 (1.1)	0.09*	13.2-17.5	13.2-15.8
*Volumes*										
OCV (cm^3^)	39.1 (11.9)	39.5 (9.9)^+^	0.92*	23.0-57.6	28.4-53.9	34.3 (11.3)	30.1 (9.4)	0.37*	19.9-46.5	20.8-44.9
PCV (cm^3^)	14.9 (9.7)	15.8 (10.8)^+^	0.84*	3.8-29.4	4.2-32.1	11.3 (6.3)	6.8 (3.4)	0.07*	4.1-20	2.7-11
VTV (cm^3^)	54.0 (17.0)	55.9 (17.1)^+^	*0.80* ^ͳ^	10.0-79.8	34.2-82.1	45.4 (13.2)	37.0 (7.0)	*0.22*ͳ	28.0-63.6	31.1-48.1
*Areas*										
OJA (cm^2^)	1.8 (1.2)	1.1 (1.2)	***0.04*** ^ͳ^	0.7-3.5	0.6-3.1	1.2 (0.9)	0.6 (0.1)	***0.02*** ^ͳ^	0.6-2.8	0.5-0.8
GA (cm^2^)	1.4 (0.8)	0.6 (0.3)	0.01*	0.4-2.8	0.2-1.1	1.1 (0.6)	0.9 (0.7)	0.42*	0.4-2.3	0.5-2.1
Acoustic Parameters of Voice	mean (standard deviation)		*p*	P5-P95%		mean (standard deviation)		*p*	P5-P95%	
	HG	PD		HG	PD	HG	PD		HG	PD
f0 (Hz)	133.9 (22.1)^+^	112.6 (12.0)++	0.01*	112.6-68.7	96.8-27.5	187.9 (32.8)	187.1 (28.3)	0.95*	145-233.1	157.5-233.2
Jitter (%)	0.3 (0.4)	0.7 (1.1)	*0.17* ^ͳ^	0.1-0.2	0.1-2.8	0.7 (1.2)	0.4 (0.8)	*0.13* ^ͳ^	0.1-1.8	0.1-2.9
Shimmer (%)	6.3 (3.5)	10.5 (7.0)	*0.11* ^ͳ^	2.3-11.5	3.5-21.6	6.6 (5.5)	6.4 (4.3)	*0.70* ^ͳ^	2.1-16.3	3.3-14.3
GNE	0.7 (0.1)^+^	0.7 (0.1)	0.49*	0.4-0.9	0.6-1.0	0.8 (0.1)	0.6 (0.1)	0.07*	0.6-1.0	0.2-1.0
Noise	1.4 (0.7)^+^	1.1 (0.6)	0.21*	0.6-2.6	0.3-2.0	0.7 (0.5)	1.5 (1.1)	0.07*	0.3-1.8	0.4-3.3
Mean Intensity (dB)	39.1 (5.5)	37.0 (3.9)	0.33*	32.8-46.7	31.3-42.1	40.9 (5.9)	40.0 (4.1)	0.72*	33.4-50.4	35.3-45.2

* Independent t-test

ͳ Mann-Whitney test

+ Difference between the sexes p < 0.05

++ Difference between the sexes p < 0.0001

**Caption:** HG = Healthy Group; PD = Parkinson’s Disease Group; OCL = Oral Cavity Length; PCL = Pharyngeal Cavity Length; VTL = Vocal Tract Length; OCV = Oral Cavity Volume; PCV = Pharyngeal Cavity Volume; VTV = Vocal Tract Volume; OJA = Oropharyngeal Junction Area; GA = Glottal Area; f0 = Fundamental Frequency; GNE = Glottal-to-Noise Excitation Ratio (Proportion Between Glottal Excitation and Noise); cm = Centimeters; Hz = Hertz; dB = Decibels

**Table 4:** Values of oropharyngeal segment geometry measures and acoustic parameters of voice stratified by age groups and results of the comparison between healthy individuals and Parkinson’s disease patients

Oropharyngeal Geometry	Age ≥ 60 years (n=25)					Age < 60 years (n=15)				
	mean (standard deviation)		*p*	P5-P95%		mean (standard deviation)		*p*	P5-P95%	
	HG (n=13)	PD (n=12)		HG	PD	HG (n=7)	PD (n=8)		HG	PD
*Lengths*										
OCL (cm)	8.1 (1.1)	8.1 (1.0)	*0.90* *	7.0-9.7	7.0-9.7	9.1 (0.9)	8.7 (0.8)	0.39*	8.1-10.5	8.0-10.1
PCL (cm)	6.8 (2.7)	5.9 (1.6)	0.32*	3.5-10.7	4.0-9.0	5.9 (1.9)	5.6 (2.1)	0.73*	3.8-8.3	3.2-8.9
VTL (cm)	14.9 (2.1)	14.0 (1.4)	*0.53* ^ͳ^	13.2-17.9	13.2-16.5	15.1 (2.2)	14.7 (2.0)	*0.48*ͳ	13.2-17.9	13.2-17.7
*Volumes*										
OCV (cm^3^)	32.6 (10.0)+	33.7 (11.5)	0.78*	18.7-45.6	21.7-52.3	44.4 (10.9)	36.4 (9.5)	0.15*	30.5-58.6	24.3-50.1
PCV (cm^3^)	13.9 (9.0)	10.8 (8.4)	*0.31* ^ͳ^	4.6-27.3	2.8-25.5	11.5 (6.8)	12.1 (10.4)	*0.81* ^ͳ^	3.1-19.6	3.2-28.2
VTV (cm^3^)	46.5 (15.4)	44.7 (16.4)	*0.72* ^ͳ^	28.7-73.1	31.1-71.5	55.7 (14.8)	49.1 (16.1)	*0.42* ^ͳ^	40.5-76.8	32.6-70.5
*Areas*										
OJA (cm^2^)	1.3 (0.9)	0.9 (1.1)^+^	***0.01*** ^ͳ^	0.6-3.1	0.5-2.5	1.7 (1.2)	0.8 (0.2)	*0.08* ^ͳ^	0.7-3.6	0.7-1.2
GA (cm^2^)	1.1 (0.6)	0.8 (0.7)	*0.14* ^ͳ^	0.4-2.2	0.4-2.0	1.5 (0.9)	0.7 (0.3)	*0.07* ^ͳ^	0.5-2.8	0.2-1.0
Acoustic Parameters of Voice	mean (standard deviation)		*p*	P5-P95%		mean (standard deviation)		*p*	P5-P95%	
	HG	PD		HG	PD	HG	PD		HG	PD
f0 (Hz)	172.1 (39.3)	159.0 (47.5)	0.46*	117.4-232.9	101.0-231.7	140.2 (30.3)	136.1 (35.6)	0.81*	114.5-186.9	98.2-185.4
Jitter (%)	0.4 (0.7)	0.8 (1.2)	*0.11* ^ͳ^	0.1-2.0	0.1-3.2	0.3 (0.2)	0.6 (1.0)	***0.002*** ^ͳ^	0.1-0.8	0.1-2.3
Shimmer (%)	6.7 (5.1)	9.3 (6.4)	*0.21* ^ͳ^	2.2-16.0	3.6-20.0	5.9 (3.4)	7.2 (5.6)	*0.81* ^ͳ^	2.2-11.0	3.2-16.3
GNE	0.8 (0.1)	0.6 (0.2)	*0.05* ^ͳ^	0.5-1.0	0.3-1.0	0.7 (0.2)	0.7 (0.1)	*0.60* ^ͳ^	0.5-1.0	0.6-1.0
Noise	1.0 (0.6)	1.5 (0.9)	*0.06* ^ͳ^	0.4-2.3	0.4-3.3	1.3 (0.8)	0.8 (0.7)	*0.28* ^ͳ^	0.4-2.5	0.2-2.0
Mean Intensity (dB)	39.7 (6.3)	38.3 (4.0)	0.52*	31.3-49.9	32.8-43.5	40.5 (4.3)	38.8 (4.7)	0.49*	34.9-46.0	33.5-45.3

* Independent t-test

ͳ Mann-Whitney test

+ Difference between the ages p < 0.05

**Caption:** HG = Healthy Group; PD = Parkinson’s Disease Group; OCL = Oral Cavity Length; PCL = Pharyngeal Cavity Length; VTL = Vocal Tract Length; OCV = Oral Cavity Volume; PCV = Pharyngeal Cavity Volume; VTV = Vocal Tract Volume; OJA = Oropharyngeal Junction Area; GA = Glottal Area; f0 = Fundamental Frequency; GNE = Glottal-to-Noise Excitation Ratio (Proportion Between Glottal Excitation and Noise); cm = Centimeters; Hz = Hertz; dB = Decibels

Regarding oropharyngeal geometry, variables related to glottal area (GA) and oropharyngeal junction area (OJA) were smaller in the PD group than in HG. No differences were found between the groups in acoustic parameters of voice (Table 2).

Stratification by sex revealed smaller area values in the PD group than in HG in both sexes, except for GA in females. Moreover, PD group males had greater oral cavity length (OCL) and oral cavity (OCV), pharyngeal cavity (PCV), and vocal tract volumes (VTV) than PD group females. Regarding acoustic parameters of voice, f0 values were lower in PD males than HG males, and lower in males than females in both groups, as expected (Table 3).

Stratification by age groups revealed differences between HG and PD subjects in OJA, which was smaller in older adults with PD. However, within the PD group, OJA was greater in older adults than non-elderly adults. Regarding acoustic parameters of voice, only jitter was greater among non-elderly adults with PD (Table 4). Moreover, HG older than 60 years had lower OCV than HG non-elderly adults.

In correlation analysis of the oropharyngeal measures, the following results were found: OCL had a moderate direct correlation with OCV, representing 45% of volume variability (p < 0.0001; r = 0.67; r^2^ = 0.45). Likewise, pharyngeal cavity length (PCL) and vocal tract length (VTL) had a moderate direct correlation with PCV (p < 0.0001; r = 0.69; r^2^ = 0.47) and VTV (p < 0.0008; r = 0.51; r^2^ = 0.25), respectively representing 47% and 25% of variability. The correlation of OCL, PCL, and VTL with the other pharyngometry measures and acoustic parameters had no statistical significance and small or low rho values.

## DISCUSSION

Regarding the smaller GA in the PD population (Tables 2 and 3), it must be pointed out that this area measured with AP does not correspond to the same area measured with imaging examinations, such as computed tomography (CT scan) or magnetic resonance^(11-16,24,25)^. GA measured with AP corresponds to the cross-sectional area by the glottis, while GA measured with imaging examinations corresponds to the space between one and the other vocal fold^(9-16,24,25)^.

Hence, this explains the results of the present study, inferring that the possible smaller interarytenoid space in PD subjects than in HG^(24)^ has corroborated this result. Considering that GA measured with AP corresponds to the cross-sectional area by the glottis while breathing^(9,10,14,15)^ (abducted vocal folds), the glottal configuration at rest interfered with such a measure. Thus, given that the smaller interarytenoid space in PD subjects^(24)^ diminishes such cross-sectional area, smaller GA in the PD group than in HG is explained.

A study whose participants were in more advanced PD stages found greater GA in them than in controls; this measure is considered a marker of disease progression^(25)^. However, two considerations must be made: 1) the said study used CT scan^(25)^, unlike the present one; 2) PD stages in the present study corroborate these results (Table 1), as greater cross-sectional GA could be expected in individuals in more advanced stages of the disease, while those in the present study were in stages 1 and 2 in HY scale.

Hence, regarding methods, the study with CT scan calculated GA as the space between vocal folds in adduction and abduction^(24,25)^. With AP, on the other hand, GA is influenced not only by the distance between vocal folds in glottal abduction but also by the whole cross-sectional area in the region.

Regarding OJA measures, likewise smaller in PD subjects, the lowered soft palate in the PD population is inferred to explain such results^(26)^. Stratification by sex shows that this measure is smaller in PD in both males and females and that only OJA was smaller in PD females. Therefore, it must be considered that, in the study group, the interference of hypokinetic dysarthrophonia had a greater impact on males.

Future studies should compare laryngeal examination data with AP to verify vocal fold positioning at rest and in phonation in this population, comparing them with GA measures. Moreover, OJA results should be compared with nasalance values in these groups, using instrumental and nasality measures and auditory-perceptual analysis.

Smaller f0 values in PD males than same-sex HG (Table 3) may be associated with stiffness in PD individuals, which is characteristic of the disease^(4)^. Considering that such stiffness can impact the function of vocal fold tensor muscles, it would decrease the possibilities of tone flexibility in voice production.

Furthermore, hypokinetic dysarthrophonia caused by PD^(24,27)^ may affect the contraction not only of the cricothyroid muscle (thus decreasing vocal fold stretch) but also of the extrinsic laryngeal musculature. Hence, in sustained emission when healthy individuals usually tend to increase voice pitch and elevate the larynx, PD patients’ larynx may remain lowered due to hypokinesia of suprahyoid muscles, which are responsible for laryngeal elevation.

Nonetheless, differences in f0 between males and females in both groups (HG and PD) were expected, as men have lower f0 than women^(28)^. Also, greater OCL, OCV, PCV, and VTV values in PD males than in PD females were likewise expected, as male VT tends to have greater dimensions than female VT^(14,29)^. Interestingly, such difference was not found in HG, allowing the inference that the sample size may have influenced this result, as well as not having controlled racial factors in participating individuals - for it is known that ethnicity influences oropharyngeal geometry measures^(29)^.

As for the results of stratification by age, smaller OJA in the PD group than in HG in the population older than 60 years may be due to possibly hypofunctioning soft palates, caused by both PD and aging - although resonance consequences were not evident in this sample^(26)^.

Lower OCV values in subjects older than 60 years than in non-elderly adults in HG may be explained by tooth loss and the consequent tendency to greater bone absorption characteristic of aging, which diminishes OCV in this group^(30)^.

As for those below 60 years old, greater jitter values in the PD group than in HG may be explained by disease characteristics. This parameter may be changed in people with neurological dysphonia for the lack of control over glottal cycles of vocal fold vibration - i.e., greater disturbance in vibratory cycles and greater vocal instability^(28)^. Despite the normal mean values, attention is drawn to such a difference between the groups; hence, future studies should compare these results with disease stages.

Regarding correlations, the association between increased OCL and OCV is explained by how the distance from the central incisors to the soft palate influences the calculation of this cavity volume. PCL and VTL likewise influence the calculation of PCV and VTV measures^(9,14,15)^.

Therefore, the present study helped identify VT segments with differences between healthy people and PD patients in the initial stages of the disease. This identification may explain possible voice deterioration in the course of the disease and may be useful in early voice treatment in PD.

Furthermore, this research reinforced the presupposition that AP improves VT assessments, whose measures can be compared with respective voice results. Hence, future studies should assess the effect of vocal techniques on oropharyngeal geometry, comparing it with voice analysis. It was also observed that AP is a quick noninvasive method that can be applied in older adults and people with neurological changes like PD. Based on the knowledge attained with this research, future studies should investigate possible associations between VT dimensions and acoustic measures of voice related to the formants^(7)^.

The limitations of this study include the number of participants, as stratifications by sex and age diminished representativity. Hence, this study should be continued with a larger sample. Further research should also implement auditory-perceptual voice assessments and voice self-assessment questionnaires, which may contribute to multidimensional analyses of voice. Nevertheless, this was an unprecedented study in a national speech-language-hearing journal addressing VT geometry measures in PD patients, using AP.

## CONCLUSION

Individuals with PD had smaller GA and OJA than healthy people. When distributed into sex and age groups, f0 was smaller in PD males, and jitter values were greater in PD non-elderly adults. There were moderate positive correlations between OCL and OCV measures, PCL and VTL measures, and PCV and VTV measures in the sample.
