# Supplementary material for: Oropharyngeal geometry and acoustic parameters of voice in healthy and Parkinson's disease subjects
Source: Codas. 2023 Apr 17;35(2):e20210304. doi: 10.1590/2317-1782/20232021304en (PMC10137768; doi:10.1590/2317-1782/20232021304en)
Supplement: Translation

# Translation: Geometria orofaríngea e parâmetros acústicos vocais de indivíduos hígidos e com doença de Parkinson

**DOI:** 10.1590/2317-1782/20232021304pt

**Authors:** Joice Maely Souza da Silva, Adriana de Oliveira Camargo Gomes, Maria das Graças Wanderley de Sales Coriolano, Julianne Pitanga Teixeira, Hellen Vasconcelos Silva Leal de Lima, Clarissa Evelyn Bandeira Paulino, Hilton Justino da Silva, Zulina Souza de Lira

**Affiliations:** 1 Programa de Pós-graduação em Saúde da Comunicação Humana, Universidade Federal de Pernambuco - UFPE - Recife (PE), Brasil.; 2 Departamento de Fonoaudiologia, Universidade Federal de Pernambuco - UFPE - Recife (PE), Brasil.; 3 Departamento de Anatomia, Universidade Federal de Pernambuco - UFPE - Recife (PE), Brasil.

**Keywords:** Orofaringe, Qualidade da Voz, Acústica, Disfonia, Doença de Parkinson

## Abstract

**Objetivo:**

verificar se existem diferenças nas medidas acústicas e da geometria orofaríngea entre indivíduos hígidos e pessoas com Doença de Parkinson, segundo a idade e sexo e investigar se há correlações entre as medidas geométricas orofaríngeas nessa população.

**Método:**

participaram 40 indivíduos, sendo 20 com diagnóstico de Doença de Parkinson e 20 indivíduos hígidos, pareados por faixa etária, sexo e índice de massa corporal. As variáveis acústicas estudadas foram frequência fundamental, *jitter*, *shimmer, glottal-to-noise excitation ratio*, ruído e média da intensidade. As variáveis geométricas da orofaringe foram aferidas por faringometria acústica.

**Resultados:**

as variáveis geométricas foram menores no grupo com Doença de Parkinson e os idosos com Doença de Parkinson apresentaram menor área da junção orofaríngea que os idosos hígidos. Com relação aos parâmetros acústicos vocais, o valor da frequência fundamental foi menor no sexo masculino, no grupo com Doença de Parkinson e os valores de *jitter* foram maiores no grupo não idoso dos sujeitos com Doença de Parkinson. Houve correlação positiva moderada entre o comprimento e volume da cavidade oral, comprimento da cavidade faríngea e o comprimento do trato vocal e do volume da cavidade faríngea e o volume do trato vocal.

**Conclusão:**

indivíduos com Doença de Parkinson apresentaram menores valores de área glótica e área da junção orofaríngea, comparativamente aos hígidos. Quando distribuídos por faixa etária e sexo, a frequência fundamental foi menor no grupo com doença de Parkinson, na população masculina. Houve correlação positiva moderada entre as medidas de comprimento e volume da orofaringe, na amostra estudada.

## INTRODUÇÃO

A Doença de Parkinson (DP) é uma enfermidade crônica, caracterizada pela degeneração da porção compacta da substância negra do mesencéfalo, acarretando na perda de neurônios dopaminérgicos^(1-3)^. Suas principais manifestações motoras incluem bradicinesia, rigidez, tremor de repouso, alterações posturais e da marcha^(3,4)^. Alterações na produção vocal, como dificuldades na coordenação pneumofonoarticulatória, diminuição da intensidade vocal, rouquidão, voz áspera, aumento da nasalidade e redução do controle muscular das estruturas laríngeas são alguns sinais que podem surgir no decurso da doença^(5)^.

No entanto, as alterações relacionadas à produção da voz, nesses indivíduos, geralmente são analisadas do ponto de vista fonatório, ou seja, relacionadas à fonte glótica e à função aerodinâmica, com poucas referências ao trato vocal (TV). Desse modo, pouco se consideram as interferências do “filtro” que atua como modificador dos padrões de vibração das pregas vocais, pois suas estruturas oferecem, em determinados segmentos, obstáculos à onda sonora gerada na fonte glótica^(6)^. Portanto, as cavidades do TV, que englobam as estruturas orofaríngeas, estão diretamente relacionadas ao som resultante da voz, de modo que as dimensões dessas estruturas impactam na qualidade vocal^(7)^.

Sabendo-se que as pessoas com DP apresentam alterações que impactam diretamente na fala e voz^(8)^, e que a produção da voz é uma composição do som produzido na glote e dos efeitos de ressonância ao longo do TV^(6)^, o estudo das dimensões do TV pode trazer respostas importantes para o diagnóstico e tratamento desses indivíduos. Como exemplo, tal estudo pode contribuir na identificação de quais segmentos do TV resultariam em aumento ou diminuição da intensidade ou projeção vocais, associando-se suas dimensões à qualidade vocal resultante.

Ademais, o acompanhamento dos diferentes ajustes do TV, por meio de técnicas terapêuticas ou de aperfeiçoamento da voz, mensurada a sua geometria e seus respectivos resultados vocais, auxiliariam no monitoramento do efeito das técnicas para a qualidade vocal pretendida. Essa hipótese corrobora a ideia de que métodos instrumentais não invasivos que permitem medir o trato vocal e os ajustes na região orofaríngea possibilitam um aprimoramento da avaliação e do acompanhamento de resultados terapêuticos na clínica vocal, quando correlacionadas à análise da voz^(9-11)^.

A análise das dimensões de área, volume e comprimento de diferentes segmentos da região orofaríngea, por exemplo, pode ser realizada por meio da faringometria acústica (FA)^(9,12)^, cujo princípio físico é o de que um som gerado em um tubo retorna em forma de eco. A intensidade mensurada no retorno desse som (eco) representa a área de secção transversal das diferentes constrições desse tubo e a distância de cada uma das constrições é calculada pelo tempo de chegada do eco permitindo um mapeamento de toda a cavidade, a partir dos incisivos até à glote^(9,10,13)^.

Além disso, a FA é um exame que permite respiração livre durante o procedimento, é rápido, de baixo custo - comparativamente aos exames de imagem - não-invasivo, e não expõe o paciente à radiação como outros exames^(14)^. Seu uso pioneiro foi em pesquisas na área do sono^(12,15-17)^, entretanto, a faringometria acústica ainda é pouco utilizada em estudos no Brasil, com recentes publicações relacionadas à voz^(9,10)^.

Quanto à análise dos parâmetros acústicos vocais, é importante ressaltar que, além de permitir maior qualidade à avaliação vocal, por apresentarem dados que não são puramente subjetivos, ou seja, que não dependem exclusivamente da experiência auditiva do avaliador, possibilita também a detecção de manifestações vocais, em quadros subclínicos, na população com doenças neurológicas, contribuindo, inclusive, com o diagnóstico dessas doenças^(18,19)^.

Portanto, ratifica-se a importância de se verificar as medidas da geometria orofaríngea e dos parâmetros acústicos da voz em sujeitos hígidos e com doença de Parkinson, considerando-se a idade e sexo dos sujeitos, bem como as possíveis correlações entre as medidas orofaríngeas. Tais resultados poderão contribuir com os estudos que visam embasar o uso da faringometria acústica no diagnóstico e monitoramento de resultados terapêuticos vocais.

Desse modo, o objetivo do presente estudo foi verificar se existem diferenças nas medidas acústicas e da geometria orofaríngea entre indivíduos hígidos e pessoas com Doença de Parkinson, segundo a idade e sexo, assim como investigar se há correlações entre as medidas geométricas orofaríngeas nessa população.

## MÉTODO

Participaram do estudo 40 indivíduos entre 50 a 70 anos, sendo 20 com diagnóstico de DP (10 homens e 10 mulheres). Para a comparação dos resultados dessa população com indivíduos sem a DP, foram incluídos 20 indivíduos hígidos (10 homens e 10 mulheres) pareados aos sujeitos com DP, de acordo com a faixa etária, sexo e índice de massa corporal (IMC). Os participantes com DP foram recrutados do ambulatório de Neurologia de um Hospital Universitário. Para compor o grupo de indivíduos hígidos, foram recrutados acompanhantes dos pacientes que frequentavam o hospital e dos que frequentavam a Clínica Escola de Fonoaudiologia da mesma Instituição, amigos e conhecidos dos sujeitos da pesquisa e que não tivessem diagnóstico de qualquer alteração neurológica ou vocal.

Desse modo, o estudo foi constituído por amostragem por conveniência, aprovado pelo Comitê de Ética em Pesquisas com Seres Humanos, sob o parecer de número 2.524.982. Todos os participantes envolvidos no estudo assinaram o termo de consentimento livre e esclarecido. Os critérios de inclusão consistiram em: pacientes com diagnóstico de doença de Parkinson, classificados considerando-se os estágios 1, 2 e 3, segundo a versão original da escala de *Hoehn &Yahr* (HY)^(20)^ e com cognição preservada, verificada pelo Mini Exame do Estado Mental (MEEM)^(21)^. Para a estratificação por faixa etária, considerou-se como idoso o indivíduo que apresentasse 60 anos ou mais^(22)^.

As informações relacionadas ao diagnóstico das doenças laríngeas foram obtidas por meio da videolaringoscopia, antes do início da coleta de dados e apenas os indivíduos que não apresentaram lesões ou más-formações laríngeas fizeram parte da pesquisa. Indivíduos com DP associada a outras comorbidades neurológicas e psiquiátricas, com relato de cirurgia laríngea, cirurgia de cabeça e pescoço, que alegavam ser tabagistas e/ou etilistas, que estivessem gripados no momento da pesquisa ou em crises alérgicas, como rinite ou sinusite, foram excluídos da pesquisa.

A caracterização da amostra, de acordo com os dois grupos estudados, apresenta a homogeneidade entre os grupos, em relação à idade, aos resultados obtidos no Mini Exame do Estado Mental, aos anos de instrução e ao índice de massa corporal (Tabela 1).

**Tabela 1:** Caracterização da amostra (n=40 indivíduos)

Grupo Hígido (n=20)	média (desvio-padrão)	valores mínimo-máximo
Idade (anos) - 13 idosos e 7 não idosos	61 (5)	50-69
Mini exame do estado mental	24 (4)	14-30
Anos de instrução	8 (5)	2-12
Índice de massa corporal	26 (2)	23-29
Grupo Doença de Parkinson (n=20)	média (desvio-padrão)	valores mínimo-máximo
Idade (anos) - 12 idosos e 8 não idosos	61 (6)	50-69
Mini exame do estado mental	27 (3)	20-30
Anos de instrução	9 (4)	4-15
Índice de massa corporal	25 (2)	21-31
Tempo de doença (anos)	7 (5)	1-20
Estágio da doença (HY)	2 (1)	1-2

**Legenda:** HY = Escala de Hoehn & Yahr^(20)^

A análise acústica ocorreu por meio de gravações dos registros vocais. Os participantes foram instruídos a sentar-se em uma cadeira confortável em ângulo de 90º e, em seguida, emitir a vogal /ɛ/ sustentada por cinco segundos e a contagem de um a 10, em voz habitual. As tarefas de fala foram gravadas com taxa de amostragem de 44000Hz, com 16 bits por amostra. A coleta realizou-se com o Notebook n3 Intel® Core™ i3-2348M, utilizando o Adaptador *Andrea PureAudio*™ USB-AS, que se trata de uma placa de som externa e microfone Auricular *Karsect* HT-2, mantendo uma distância de, aproximadamente, quatro centímetros da boca, em um ângulo de aproximadamente 45º.

Os dados acústicos foram registrados e editados no software Voxmetria® da CTS informática. O segundo inicial e o segundo final da gravação da vogal sustentada foram eliminados, buscando-se excluir os trechos de maior irregularidade da amostra, conservando o tempo de três segundos de emissão, para análise. Os dados relativos à frequência fundamental (f0), *jitter e shimmer, glottal-to-noise excitation ratio* (GNE) e ruído foram extraídos a partir da emissão da vogal /ɛ/ e a média da intensidade foi obtida a partir da contagem de números. Todos os parâmetros foram calculados pelo *software* Voxmetria®.

Todos os participantes foram submetidos à avaliação da geometria orofaríngea por meio da faringometria acústica, em estado de vigília. Para tal, utilizou-se o faringômetro acústico da marca *Eccovision*® - *Sleep Group Solutions*, Flórida, instalado no Laboratório da instituição de origem deste trabalho, sendo controlada a temperatura (25º) e o ruído (inferior a 60dB NPS) durante a realização do exame.

Para mensuração das medidas da orofaringe e trato vocal, o faringômetro foi calibrado automaticamente^(9,10)^. Os participantes permaneceram sentados, em uma cadeira com encosto, apresentando postura de cabeça e tronco alinhados. Foram instruídos a morder a boquilha de material plástico e selar com os lábios ao redor, impedindo o vazamento acústico. Essa boquilha é conectada ao faringômetro em uma extremidade, posicionada horizontalmente ao examinador e paralelo ao solo. Visando manter a postura, o participante foi orientado a fixar o olhar em um ponto à sua frente, mantendo o fluxo de ar habitual.

Para cada medida, o programa (*software*) gerou um gráfico da relação distância (eixo das coordenadas) e área (eixo das abscissas), subdividido em três regiões: oral (dos incisivos até o palato mole), faríngea (do palato mole até à hipofaringe) e laríngea (região glótica).

Os participantes foram instruídos a inspirar de forma habitual, sempre pelo nariz por alguns instantes e, logo em seguida, assim que acordado com o pesquisador, realizava-se a expiração oral, sendo o final de cada expiração o momento da captura das medidas^(9,10)^.

Foram realizadas quatro medidas exibidas em quatro janelas no monitor do equipamento, a saber:

- Medidas da região orofaríngea (registradas nas duas primeiras janelas): a orientação aos participantes consistiu em inspirar pelo nariz e expirar lentamente pela boca. Os gráficos serviram de base para as medidas orofaríngeas por se caracterizar como possíveis gráficos calibradores, apresentando-se de forma sobreposta e com máximo percentual de reprodutibilidade, aceitando-se variação de até 6%.
- Medida da junção orofaríngea (registrada na terceira janela): os participantes foram orientados a inspirar e expirar o ar pelo nariz. Seguindo essa instrução foi possível identificar a junção orofaríngea, delimitada ao final da cavidade oral, quando o véu palatino se apresenta abaixado.
- Medida da região glótica (registrada na quarta janela): a orientação aos participantes consistiu na inspiração pelo nariz e realização da manobra de Valsalva, na qual o indivíduo foi instruído a tampar as narinas com os dedos e em seguida fazer força, promovendo o fechamento glótico. Dessa forma, possibilitou-se localizar, no gráfico, o final da cavidade faríngea, indicando a região da glote.

Os dados foram compilados e expressos por medidas de tendência central e dispersão. Para verificar a normalidade das séries de dados foi realizado Teste de Shapiro-Wilk que determinou os testes de comparação entre as médias dos grupos (Teste T independente ou Mann-Whitney). A correlação entre as variáveis foi realizada pelo Teste de Pearson, cuja interpretação seguiu o seguinte critério: 0,90 a 1,00 = “Muito alta”; 0,70 a 0,90 = “Alta”; 0,50 a 0,70 = “Moderada”; 0,30 a 0,50 = “Baixa”; 0,10 a 0,30 = “Pequena”^(23)^. Além do *rho* de Pearson, também foram expressos os coeficientes de determinação, r^2^. O pacote estatístico utilizado foi o *Statistica StatSoft* 12, considerando-se como significantes os valores de p<0,05.

## RESULTADOS

Os valores médios das medidas da geometria orofaríngea e dos parâmetros acústicos vocais estão expressos nas Tabelas 2, 3 e 4, bem como a comparação entre os grupos DP e hígido. Na Tabela 3, as medidas foram estratificadas por sexo e na Tabela 4, foram estratificadas por faixa etária.

**Tabela 2:** Valores das medidas da geometria dos segmentos orofaríngeos e dos parâmetros acústicos vocais dos sujeitos hígidos e com doença de Parkinson e resultados da comparação entre os dois grupos

Geometria Orofaríngea	Grupo Hígido (n=20)			Grupo Doença de Parkinson (n=20)			
	média (desvio-padrão)	*range*	P5-P95%	média (desvio-padrão)	*range*	P5-P95%	*p-valor*
*Comprimentos*							
CCO (cm)	8,5 (1,1)	6,7-11,0	7,1-10,6	8,4 (0,9)	6,7-10,5	7,1-10,2	*0,79* *
CCF (cm)	6,5 (2,5)	2,2-10,7	3,3-10,7	5,8 (1,8)	2,6-9,4	3,0-9,0	*0,67*ͳ
CTV (cm)	15,0 (2,1)	13,1-17,8	13,2-17,9	14,3 (1,7)	13,1-17,8	13,2-17,9	*0,37* ^ͳ^
*Volumes*							
VCO (cm^3^)	36,7 (11,6)	17,0-60,6	19,7-54,3	34,8 (10,6)	20,2-54,0	21,3-53,8	0,59*
VCF (cm^3^)	13,1 (8,2)	2,4-36,3	3,7-22,0	11,3 (9,1)	2,2-34,6	3,0-29,2	*0,33* ^ͳ^
VTV (cm^3^)	49,7 (15,5)	26,1-81,3	30,1-78,0	46,4 (16)	31,0-88,6	31,1-74,8	*0,39* ^ͳ^
*Áreas*							
AJO (cm^2^)	1,5 (1,0)	0,5-3,9	0,7-3,6	0,9 (0,8)	0,4-4,6	0,5-1,5	***<0,01*** ^ͳ^
AG (cm^2^)	1,3 (0,7)	0,3-2,9	0,4-2,6	0,8 (0,5)	0,05-2,9	0,3-1,2	***0,04*** ^ͳ^
Parâmetros Acústicos Vocais	média (desvio-padrão)	*range*	P5-P95%	média (desvio-padrão)	*range*	P5-P95%	*p-valor*
f0 (Hz)	160,9 (38,8)	111,3-233,4	113,8-232,6	149,9 (43,7)	94,2-239,9	99,6-225,7	0,40*
*Jitter* (%)	0,41 (0,65)	0,09-2,86	0,1-1,5	0,78 (1,14)	0,10-4,21	0,1-3,3	*0,06* ^ͳ^
*Shimmer* (%)	6,47 (4,5)	1,8-20,0	1,9-13,7	8,54 (6,0)	3,1-23,3	3,3-19,7	*0,26* ^ͳ^
GNE	0,78 (0,17)	0,40-0,98	0,5-1,0	0,72 (0,20)	0,24-0,95	0,3-1,0	*0,25* ^ͳ^
Ruído	1,12 (0,72)	0,33-2,73	0,4-2,4	1,29 (0,89)	0,11-3,40	0,4-3,3	*0,51* ^ͳ^
MED_INT (dB)	40,0 (5,6)	31,2-52,0	31,3-48,6	39,5 (4,2)	30,2-45,4	32,4-45,0	0,35*

* Teste T independente - nível de significância de 5%

ͳ Teste de Mann-Whitney - nível de significância de 5%

**Legenda:** CCO = Comprimento da Cavidade Oral; CCF = Comprimento da Cavidade Faríngea; CTV = Comprimento do Trato Vocal; VCO = Volume da Cavidade Oral; VCF = Volume da Cavidade Faríngea; VTV = Volume do Trato Vocal; AJO = Área da Junção Orofaríngea; AG = Área Glótica; f0 = Frequência Fundamental; GNE = *Glottal to Noise Excitation Ratio* (Proporção da Excitação Glótica Sobre o Ruído); MED INT = Intensidade Média; *range* = Valores Máximo-Mínimo; cm = Centímetros; Hz = Hertz; dB = Decibels

**Tabela 3:** Valores das medidas da geometria dos segmentos orofaríngeos e dos parâmetros acústicos vocais, estratificados por sexo, e os resultados da comparação entre os indivíduos hígidos e com doença de Parkinson

Geometria orofaríngea	Sexo Masculino (n=20)					Sexo Feminino (n=20)				
	média (desvio-padrão)		*p-valor*	P5-P95%		média (desvio-padrão)		*p-valor*	P5-P95%	
	GH (n=10)	DP (n=10)		GH	DP	GH (n=10)	DP (n=10)		GH	DP
*Comprimentos*										
CCO (cm)	8,7 (1,1)	8,8 (0,7)+	*0,87* *	7,2-10,4	8,2-10,0	8,2 (1,1)	8,0 (0,9)	0,58*	7,1-10,2	6,9-9,4
CCF (cm)	6,3 (2,6)	6,2 (2,1)	0,88*	3,8-10,7	3,4-9,2	6,7 (2,4)	5,4 (0,4)	0,14*	3,3-9,7	3,8-7,5
CTV (cm)	15,0 (2,4)	15,0 (2,0)	0,85*	13,2-17,9	13,2-17,9	14,9 (1,9)	13,6 (1,1)	0,09*	13,2-17,5	13,2-15,8
*Volumes*										
VCO (cm^3^)	39,1 (11,9)	39,5 (9,9)^+^	0,92*	23,0-57,6	28,4-53,9	34,3 (11,3)	30,1 (9,4)	0,37*	19,9-46,5	20,8-44,9
VCF (cm^3^)	14,9 (9,7)	15,8 (10,8)^+^	0,84*	3,8-29,4	4,2-32,1	11,3 (6,3)	6,8 (3,4)	0,07*	4,1-20	2,7-11
VTV (cm^3^)	54,0 (17,0)	55,9 (17,1)^+^	*0,80* ^ͳ^	10,0-79,8	34,2-82,1	45,4 (13,2)	37,0 (7,0)	*0,22* ^ͳ^	28,0-63,6	31,1-48,1
*Áreas*										
AJO (cm^2^)	1,8 (1,2)	1,1 (1,2)	***0,04***ͳ	0,7-3,5	0,6-3,1	1,2 (0,9)	0,6 (0,1)	***0,02*** ^ͳ^	0,6-2,8	0,5-0,8
AG (cm^2^)	1,4 (0,8)	0,6 (0,3)	0,01*	0,4-2,8	0,2-1,1	1,1 (0,6)	0,9 (0,7)	0,42*	0,4-2,3	0,5-2,1
Parâmetros Acústicos Vocais	média (desvio-padrão)		*p*	P5-P95%		média (desvio-padrão)		*p*	P5-P95%	
	GH	DP		GH	DP	GH	DP		GH	DP
f0 (Hz)	133,9 (22,1)^+^	112,6 (12,0)++	0,01*	112,6-68,7	96,8-27,5	187,9 (32,8)	187,1 (28,3)	0,95*	145-233,1	157,5-233,2
*Jitter* (%)	0,3 (0,4)	0,7 (1,1)	*0,17* ^ͳ^	0,1-0,2	0,1-2,8	0,7 (1,2)	0,4 (0,8)	*0,13* ^ͳ^	0,1-1,8	0,1-2,9
*Shimmer* (%)	6,3 (3,5)	10,5 (7,0)	*0,11* ^ͳ^	2,3-11,5	3,5-21,6	6,6 (5,5)	6,4 (4,3)	*0,70* ^ͳ^	2,1-16,3	3,3-14,3
GNE	0,7 (0,1)^+^	0,7 (0,1)	0,49*	0,4-0,9	0,6-1,0	0,8 (0,1)	0,6 (0,1)	0,07*	0,6-1,0	0,2-1,0
Ruído	1,4 (0,7)^+^	1,1 (0,6)	0,21*	0,6-2,6	0,3-2,0	0,7 (0,5)	1,5 (1,1)	0,07*	0,3-1,8	0,4-3,3
MED_INT (dB)	39,1 (5,5)	37,0 (3,9)	0,33*	32,8-46,7	31,3-42,1	40,9 (5,9)	40,0 (4,1)	0,72*	33,4-50,4	35,3-45,2

* Teste T independente

ͳ Teste de Mann-Whitney

+ Diferença entre os sexos p<0,05

++ Diferença entre os sexos p<0,0001

**Legenda:** GH = Grupo Hígido; DP = Grupo com Doença de Parkinson; CCO = Comprimento da Cavidade Oral; CCF = Comprimento da Cavidade Faríngea; CTV = Comprimento do Trato Vocal; VCO = Volume da Cavidade Oral; VCF = Volume da Cavidade Faríngea; VTV = Volume do Trato Vocal; AJO = Área da Junção Orofaríngea; AG = Área Glótica; f0 = Frequência Fundamental; GNE = *Glottal to Noise Excitation Ratio* (Proporção da Excitação Glótica Sobre o Ruído); MED INT = Intensidade Média; cm = Centímetros; Hz = Hertz; dB = Decibels

**Tabela 4:** Valores das medidas da geometria dos segmentos orofaríngeos e dos parâmetros acústicos vocais e os resultados da comparação entre os indivíduos hígidos e com doença de Parkinson, estratificados por faixa etária

Geometria orofaríngea	Idade ≥ 60 anos (n=25)					Idade < 60 anos (n=15)				
	média (desvio-padrão)		*p*	P5-P95%		média (desvio-padrão)		*p*	P5-P95%	
	GH (n=13)	DP (n=12)		GH	DP	GH (n=7)	DP (n=8)		GH	DP
*Comprimentos*										
CCO (cm)	8,1 (1,1)	8,1 (1,0)	*0,90* *	7,0-9,7	7,0-9,7	9,1 (0,9)	8,7 (0,8)	0,39*	8,1-10,5	8,0-10,1
CCF (cm)	6,8 (2,7)	5,9 (1,6)	0,32*	3,5-10,7	4,0-9,0	5,9 (1,9)	5,6 (2,1)	0,73*	3,8-8,3	3,2-8,9
CTV (cm)	14,9 (2,1)	14,0 (1,4)	*0,53*ͳ	13,2-17,9	13,2-16,5	15,1 (2,2)	14,7 (2,0)	*0,48* ^ͳ^	13,2-17,9	13,2-17,7
*Volumes*										
VCO (cm^3^)	32,6 (10,0)+	33,7 (11,5)	0,78*	18,7-45,6	21,7-52,3	44,4 (10,9)	36,4 (9,5)	0,15*	30,5-58,6	24,3-50,1
VCF (cm^3^)	13,9 (9,0)	10,8 (8,4)	*0,31* ^ͳ^	4,6-27,3	2,8-25,5	11,5 (6,8)	12,1 (10,4)	*0,81* ^ͳ^	3,1-19,6	3,2-28,2
VTV (cm^3^)	46,5 (15,4)	44,7 (16,4)	*0,72* ^ͳ^	28,7-73,1	31,1-71,5	55,7 (14,8)	49,1 (16,1)	*0,42* ^ͳ^	40,5-76,8	32,6-70,5
*Áreas*										
AJO (cm^2^)	1,3 (0,9)	0,9 (1,1)^+^	***0,01*** ^ͳ^	0,6-3,1	0,5-2,5	1,7 (1,2)	0,8 (0,2)	*0,08* ^ͳ^	0,7-3,6	0,7-1,2
AG (cm^2^)	1,1 (0,6)	0,8 (0,7)	*0,14* ^ͳ^	0,4-2,2	0,4-2,0	1,5 (0,9)	0,7 (0,3)	*0,07* ^ͳ^	0,5-2,8	0,2-1,0
Parâmetros Acústicos Vocais	média (desvio-padrão)		*p*	P5-P95%		média (desvio-padrão)		*p*	P5-P95%	
	GH	DP		GH	DP	GH	DP		GH	DP
f0 (Hz)	172,1 (39,3)	159,0 (47,5)	0,46*	117,4-232,9	101,0-231,7	140,2 (30,3)	136,1 (35,6)	0,81*	114,5-186,9	98,2-185,4
*Jitter* (%)	0,4 (0,7)	0,8 (1,2)	*0,11* ^ͳ^	0,1-2,0	0,1-3,2	0,3 (0,2)	0,6 (1,0)	***0,002*** ^ͳ^	0,1-0,8	0,1-2,3
*Shimmer* (%)	6,7 (5,1)	9,3 (6,4)	*0,21* ^ͳ^	2,2-16,0	3,6-20,0	5,9 (3,4)	7,2 (5,6)	*0,81* ^ͳ^	2,2-11,0	3,2-16,3
GNE	0,8 (0,1)	0,6 (0,2)	*0,05* ^ͳ^	0,5-1,0	0,3-1,0	0,7 (0,2)	0,7 (0,1)	*0,60* ^ͳ^	0,5-1,0	0,6-1,0
Ruído	1,0 (0,6)	1,5 (0,9)	*0,06* ^ͳ^	0,4-2,3	0,4-3,3	1,3 (0,8)	0,8 (0,7)	*0,28* ^ͳ^	0,4-2,5	0,2-2,0
MED_INT (dB)	39,7 (6,3)	38,3 (4,0)	0,52*	31,3-49,9	32,8-43,5	40,5 (4,3)	38,8 (4,7)	0,49*	34,9-46,0	33,5-45,3

* Teste T independente

ͳ Teste de Mann-Whitney

+ Diferença entre as idades p<0,05

**Legenda:** GH = Grupo Hígido; DP = Grupo com Doença de Parkinson; CCO = Comprimento da Cavidade Oral; CCF = Comprimento da Cavidade Faríngea; CTV = Comprimento do Trato Vocal; VCO = Volume da Cavidade Oral; VCF = Volume da Cavidade Faríngea; VTV = Volume do Trato Vocal; AJO = Área da Junção Orofaríngea; AG = Área Glótica; f0 = Frequência Fundamental; GNE = *Glottal to Noise Excitation Ratio* (Proporção da Excitação Glótica Sobre o Ruído); MED INT = Intensidade Média; cm = Centímetros; Hz = Hertz; dB = Decibels

Quanto às medidas da geometria orofaríngea, as variáveis relacionadas às áreas glótica (AG) e da junção orofaríngea (AJO) foram menores no grupo DP, comparativamente ao grupo de indivíduos hígidos (GH). Com relação aos parâmetros acústicos vocais, não foram observadas diferenças entre os grupos (Tabela 2).

Quando estratificados por sexo, os valores das áreas foram menores no grupo DP comparativamente ao GH, em ambos os sexos, exceto na área da glote (AG) no sexo feminino. Ademais, o grupo DP masculino apresentou valores do comprimento da cavidade oral (CCO) e dos volumes (VCO, VCF e VTV), maiores que o grupo DP feminino. Com relação aos parâmetros acústicos vocais, o valor de f0 foi menor no grupo DP, comparativamente ao GH, no sexo masculino e o valor da f0 foi menor no sexo masculino que no sexo feminino, em ambos os grupos, como esperado (Tabela 3).

Ao se estratificar os grupos por faixa etária, observou-se diferença entre os idosos do GH e DP em relação à área da junção orofaríngea (AJO) que foi menor na população idosa com DP. Ao comparar o grupo DP idoso e o não-idoso observou-se maior AJO nos idosos com DP. Com relação aos parâmetros acústicos vocais, apenas o *jitter* foi maior no grupo DP não idoso (Tabela 4). Além disso, o grupo hígido com mais de 60 anos apresentou menor VCO do que o grupo hígido dos não idosos.

Quanto à análise de correlação das medidas orofaríngeas, os resultados encontrados foram: o comprimento da cavidade oral (CCO) apresentou correlação moderada direta com o volume da cavidade oral (VCO) representando 45% da variabilidade do volume (p<0,0001; r=0,67; r^2^=0,45). Do mesmo modo, o comprimento da cavidade faríngea (CCF) e o comprimento do trato vocal (CTV) apresentaram correlação moderada direta com o volume da cavidade faríngea (P<0,0001; r=0,69; r^2^=0,47) e volume do trato vocal (P<0,0008; r=0,51; r^2^=0,25), representando 47% e 25% da variabilidade, respectivamente. A correlação do CCO, CCF e CTV com as demais medidas faringométricas e com os parâmetros acústicos não apresentou significância estatística e valor de *rho* pequeno ou baixo.

## DISCUSSÃO

Quanto às medidas de AG menores na população com Parkinson (Tabelas 2 e 3), destaca-se que essa área, medida por faringometria acústica, não corresponde à mesma medida aferida por exames de imagem, como a tomografia computadorizada ou ressonância magnética, por exemplo^(11-16,24,25)^. É importante ter em vista que a AG, mensurada na FA, corresponde à área de secção transversal no nível da glote, enquanto que as medidas de área da glote, nos exames de imagem, correspondem ao espaço entre uma prega vocal e outra^(9-16,24,25)^.

Desse modo, pode-se justificar os resultados encontrados no presente estudo, inferindo-se que a possível menor distância interaritenoidea nos sujeitos com DP, comparativamente ao grupo controle^(24)^ tenha corroborado esse resultado. Considerando-se que a AG, aferida pela FA, corresponde à medida de área de secção transversal no nível da glote durante a respiração^(9,10,14,15)^ (pregas vocais em abdução) tal medida sofre interferência da configuração glótica, em repouso. Portanto, tendo-se em conta que a menor distância entre as aritenoides, nos sujeitos com DP^(24)^ diminua essa área seccional, justifica-se o menor valor de AG no grupo com DP, comparativamente ao grupo hígido.

Vale ressaltar que, em estudo com participantes em estágios mais avançados da DP foram observadas maiores medidas de AG, comparativamente aos controles, sendo essa medida apontada como um marcador de progressão da doença^(25)^. Desse modo, duas considerações podem ser feitas: 1) o estudo citado foi realizado por meio de tomografia computadorizada (TC)^(25)^ diferentemente do presente estudo; 2) o estágio da doença, no grupo DP do presente estudo corrobora tais resultados (Tabela 1), visto que poderia se esperar maior área seccional na região glótica em indivíduos em estágios mais avançados da doença e, no presente estudo, os sujeitos apresentavam-se nos estágios 1 e 2 da escala de HY.

Portanto, quanto ao método utilizado, há que se considerar que, no estudo com TC, a área glótica calculada corresponde ao espaço entre as pregas vocais, nos momentos de adução e abdução^(24,25)^. Porém, na FA, a AG sofre influência não somente da distância entre as pregas vocais, no momento de abdução glótica, mas de toda a área seccional dessa região.

Com respeito às medidas de AJO, também menores nos sujeitos com DP, infere-se que o posicionamento do véu palatino mais rebaixado, na população com DP, justifique tais resultados^(26)^. Ao se estratificar por sexo, observa-se que tais medidas são menores, no grupo DP, tanto no sexo masculino quanto no feminino, e que apenas a AJO foi menor no sexo feminino com DP. Desse modo, há que se considerar que, no grupo estudado, a interferência do quadro de disartrofonia hipocinética tenha sido mais impactante no sexo masculino.

Em estudos futuros, recomenda-se comparar os dados do exame laríngeo aos da FA, para se verificar o posicionamento das pregas vocais em repouso e durante a fonação, nessa população, comparando-o às medidas da AG. Ademais, sugere-se comparar os resultados da AJO a valores de nasalância, por meio de medidas instrumentais e de nasalidade, por meio da análise perceptivo-auditiva, nesses grupos.

Em relação aos menores valores de f0, na população masculina com DP comparado ao grupo hígido do mesmo sexo (Tabela 3), esse achado pode estar associado ao fato de que os indivíduos com DP apresentam uma rigidez característica da doença ^(4)^. Considerando-se que essa rigidez possa impactar a função dos músculos tensores das pregas vocais, isso acarretaria menor possibilidade de flexibilidade tonal na produção da voz.

Somado a isso, há que se considerar que a disartrofonia hipocinética causada pela DP^(24,27)^ poderia afetar não apenas a contração do músculo cricotireóideo, diminuindo assim, o estiramento das pregas vocais, como pode afetar, também, a musculatura extrínseca da laringe. Portanto, pode-se inferir que, na emissão sustentada, quando geralmente indivíduos hígidos tendem a aumentar o *pitch* vocal, elevando a laringe, nos pacientes com DP a laringe pode manter-se em posição mais baixa, pela hipocinesia dos músculos supra-hioideos, responsáveis pela elevação laríngea.

No entanto, a diferença na f0 entre os sujeitos masculinos e femininos, nos dois grupos (GH e DP) era esperada, pois os homens apresentam frequências fundamentais mais baixas que as mulheres^(28)^. Ademais, os valores maiores de CCO, VCO, VCF e VTV no grupo DP masculino comparativamente aos do grupo DP feminino, também eram esperados, visto que o TV masculino tende a ter dimensões maiores que o TV feminino^(14,29)^. Curiosamente, no GH tal diferença não foi encontrada, inferindo-se que o tamanho da amostra pode ter influenciado tal resultado, ou ainda, o fato de não se ter controlado o fator racial dos indivíduos participantes, sabendo-se que a etnia pode influenciar as medidas geométricas orofaríngeas^(29)^.

Com relação aos resultados a partir da estratificação pela idade, a AJO menor no grupo DP comparativamente ao GH, na população com mais de 60 anos, pode-se atribuir a esses resultados o possível hipofuncionamento do véu palatino, atribuído tanto à doença de Parkinson, quanto ao envelhecimento, ainda que as repercussões ressonantais não estivessem evidentes nessa amostra^(26)^.

No que se refere ao menor valor no VCO no GH com mais de 60 anos de idade, comparativamente aos não idosos, esses achados podem ser explicados pela perda de elementos dentários e consequente tendência à maior absorção óssea, caracterizada pelo envelhecimento, diminuindo, assim, o volume da cavidade oral nesse grupo^(30)^.

Pela ótica da faixa etária, os valores maiores do *jitter* no grupo DP comparado ao GH na faixa etária abaixo dos 60 anos, pode-se explicar tal resultado pela própria característica da doença, tendo em vista que esse parâmetro pode estar alterado na população com disfonias neurológicas por apresentarem uma ausência de controle nos ciclos glóticos de vibração das pregas vocais, ou seja, maior perturbação nos ciclos vibratórios e maior instabilidade vocal^(28)^. A despeito da média dos valores estar dentro dos limites de normalidade, chama-se a atenção para essa diferença entre os grupos e sugere-se que em estudos futuros esses resultados sejam comparados com os estágios da doença.

Quanto às correlações encontradas, justifica-se a associação entre o aumento do comprimento da cavidade oral (CCO) e o volume da cavidade oral (VCO) pela influência do espaço delimitado pela distância dos incisivos centrais até o véu do palato, no cálculo do volume dessa cavidade. Do mesmo modo, o comprimento da cavidade faríngea (CCF) e o comprimento do trato vocal (CTV), influenciam no cálculo das medidas dos volumes da cavidade faríngea e do trato vocal^(9,14,15)^.

Destarte, pode-se considerar que o presente estudo pôde contribuir na identificação dos segmentos do TV que apresentam diferenças entre pessoas hígidas e pessoas com DP, ainda em estágios iniciais da doença. Tal identificação pode explicar possíveis deteriorações da voz no desenvolvimento da doença e pode ser útil no tratamento vocal precoce da DP.

Ademais, esta pesquisa reforçou o pressuposto de que a FA permite o aprimoramento da avaliação do TV, cujas medidas podem ser comparadas aos respectivos resultados vocais. Sugere-se, portanto, estudos que avaliem o efeito das técnicas vocais na geometria orofaríngea, comparando-a com a análise da voz. Também foi possível observar que a FA é um método rápido e não invasivo que pode ser aplicada em idosos e sujeitos com alterações neurológicas como a DP. Com base no conhecimento gerado nesta pesquisa, sugere-se a investigação de possíveis associações entre as dimensões do trato vocal e medidas acústicas da voz relativas aos formantes^(7)^, em estudos posteriores.

Como limitações deste estudo, destaca-se o número de participantes que, ao se estratificar por sexo e idade, diminui-se a representatividade; desse modo, sugere-se a continuidade deste estudo, com o aumento da amostra. Ademais, pode-se considerar a implementação das avaliações perceptivo-auditivas da voz e questionários de autoavaliação vocal que contribuiriam com a análise multidimensional da voz. No entanto, enfatiza-se o ineditismo deste estudo com medidas geométricas do trato vocal, em pacientes com Parkinson, usando-se a faringometria acústica, em revista nacional na área da Fonoaudiologia.

## CONCLUSÃO

Indivíduos com DP apresentaram menor área glótica e da junção orofaríngea, comparativamente a indivíduos hígidos. Quando distribuídos por faixa etária e sexo, a frequência fundamental foi menor no grupo com doença de Parkinson, na população masculina e os valores de *jitter* foram maiores no grupo não idoso dos sujeitos com doença de Parkinson. Houve correlação positiva moderada entre as medidas de comprimento e volume da cavidade oral e entre as medidas de comprimento da cavidade faríngea e do trato vocal e os volumes da cavidade faríngea e do trato vocal, na amostra estudada.
